# Symptom Structure of Depression in Older Adults on the Qinghai–Tibet Plateau: A Network Analysis

**DOI:** 10.3390/ijerph192113810

**Published:** 2022-10-24

**Authors:** Buzohre Eli, Yueyue Zhou, Yaru Chen, Xin Huang, Zhengkui Liu

**Affiliations:** 1 CAS Key Laboratory of Mental Health, Institute of Psychology, Chinese Academy of Sciences, Beijing 100101, China; 2 Department of Psychology, University of Chinese Academy of Sciences, Beijing 100049, China; 3 Department of Psychology, Henan University, Kaifeng 475004, China

**Keywords:** depression, older adults, high-altitude areas, network analysis, Qinghai–Tibet Plateau

## Abstract

Previous studies have confirmed that depression among residents in high-altitude areas is more severe, and that depression may be more persistent and disabling in older adults. This study aims to identify the symptom structure of depression among older adults on the Qinghai–Tibet Plateau (the highest plateau in the world) from a network perspective. This cross-sectional study enrolled 507 older adults (ages 60–80 years old) from the Yushu Prefecture, which is on the Qinghai–Tibet Plateau, China. Depressive symptoms were self-reported using the shortened Center for Epidemiological Studies–Depression Scale (CES-D-10). Then, a Gaussian graphical model (GGM) of depression was developed. Poor sleep, fear, and hopelessness about the future exhibited high centrality in the network. The strongest edge connections emerged between unhappiness and hopelessness about the future, followed by hopelessness about the future and fear; hopelessness about the future and poor sleep; fear and unhappiness; and then poor sleep and unhappiness in the network. The findings of this current study add to the small body of literature on the network structure and complex relationships between depressive symptoms in older adults in high-altitude areas.

## 1. Introduction

In recent years, living in high-altitude areas has been established as one of the risk factors for depression, mainly due to their geographic barriers, as well as the demographic and socioeconomic inequalities of high-altitude areas [1,2]. Empirical studies have indicated that depression among residents in high-altitude areas is more severe [2], and the probable depression prevalence was as high as 29.2% among residents living in high-altitude areas [3,4]. Previous studies have also indicated that the prevalence of depression increases with increasing altitude [1,5]. The harsh geographical environment and poor living conditions in high-altitude areas could also affect a person’s mental state [6,7]. Chronic hypobaric hypoxia and changes in atmospheric pressure in high-altitude areas could reduce serotonin levels in the brain [8], and thus limit the ability to regulate emotions [7], which may further increase the risk of depression. Furthermore, the economic income level is lower in high-altitude areas [9], and the poor living conditions and economic situation at high altitudes also exacerbate the vulnerability of residents to depression [6]. A study found that low income imposed a stronger risk in older adults’ depression [10]. Therefore, older adults in high-altitude areas may be more likely to suffer from depression because of their physical and mental weaknesses.

Numerous researchers have confirmed that depression may be more persistent and disabling in older adults [11,12], although depression is a common psychiatric disorder across the age range. Depression in older age is associated with high mortality [13] and poor mental health [11]. However, the mental health service system in high-altitude areas is incomplete, such as a lack of resources and professionally trained staff; further, the availability of and access to quality mental health care is limited [14]. Additionally, older adults generally have low awareness of mental health, and there are also some social prejudices and discrimination for mental problems that they may possess. Thus, they are unwilling to consult with professionals, even if they suffer from psychological problems, and they often easily miss the best psychological counselling and treatment period. This is more common among older adults in high-altitude areas. Therefore, research on depression among older adults in high-altitude areas is of great importance.

However, there is relatively little research evidence regarding depression among older adults in high-altitude areas [2,15]. These studies were focused on overall depressive symptoms, such as the frequency or severity of depression, and the most common method was to calculate the sum score [2,15]. Using the sum score of depression comes from the common cause perspective, which assumes that symptoms are passive indicators of disease and are caused by an underlying latent entity [16]. The common cause perspective also assumes that symptoms are interchangeable in the same condition, and the clustering of symptoms is due to an underlying, common cause [16,17]. The common cause view has been challenged by the network framework, i.e., that depression and other mental disorders are suggested to develop due to the self-reinforcing interactions among symptoms rather than as a result of underlying, common cause [18,19].

In this framework’s network, symptoms are represented as nodes, and the associations between these symptoms are represented as edges. Mental disorders arise from dynamic causal interactions and feedback loops (edges), among its defining symptoms (nodes) [18]. Overall, network analysis helps to identify the unique role of each individual symptom and may yield important information about the maintenance and structure of depression by revealing the most central symptoms and basic structural features of depression [20,21].

Recently, a number of studies have utilized network approaches in order to reveal the different central symptoms regarding depression. However, these studies have tended to focus on either adolescents [22,23,24] or young adults [25,26,27]; therefore, little attention has been devoted to depression in older adults [28]. A previous study revealed different high central symptoms in different samples, suggesting that replicability of network results may be limited [29]. Therefore, conclusions from previous studies may be difficult to generalize to older adults. As such, more empirical studies are needed in order to explore the network characteristics of depression in older adults living in high-altitude areas.

To address these gaps in the literature, the current cross-sectional study aims to explore the network structure of depression in older adults living in high-altitude areas. We expect to identify symptoms that play a vital role in the maintenance of depression. The Qinghai–Tibet Plateau is the largest plateau in China and also the highest plateau in the world, with an average altitude of more than 4200 m (13,776 feet). It is therefore known as the “roof of the world”. The Yushu Tibetan Autonomous Prefecture is located in the eastern hinterland of the Qinghai–Tibet Plateau. It has a harsh geographical environment, inconvenient transportation, a scattered population, and a relatively low education level among farmers and herdsmen. As of 2020, 425,199 people resided in the Qinghai–Tibetan Plateau. Of these, approximately 31,677 were older adults aged 60 years old and older, according to the data of the Qinghai Provincial Seventh National Population Census [30]. The harsh natural environment, inconvenient transportation, and poor living conditions of these areas may cause a negative impact on people′s mental health; further, older adults may be more likely to be vulnerable due to their physical and mental weaknesses. Therefore, we chose older adults in the Yushu Prefecture as the research subjects.

## 2. Materials and Methods

### 2.1. Participants

**Figure 1:**
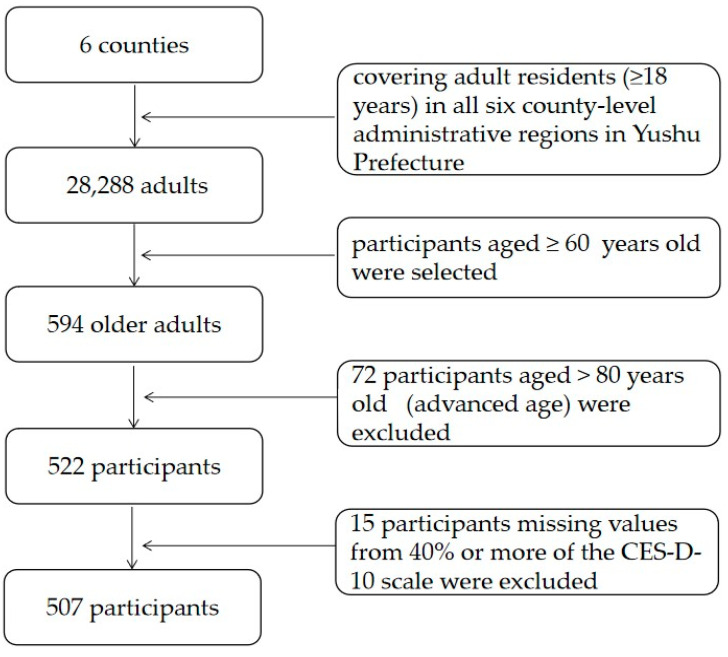
Flow chart of sampling.

This study utilized data from a large-scale cross-sectional survey, which was conducted from September to November 2016 in the Yushu Tibetan Autonomous Prefecture on the Qinghai–Tibet Plateau, China. There are six county-level administrative regions in the Yushu Prefecture (i.e., Nangqian County, Qumalai County, Zaduo County, Zhidoi County, Yushu County, and Chindu County). All adult residents in the six county-level administrative regions in the Yushu Prefecture were selected by cluster sampling and convenience sampling. The original sample consisted of 28,288 adults aged 18 years old and over. As stated in the introduction section, the current study aims to explore the network structure of depression in older adults living in high-altitude areas. This choice was made due to older adults in high-altitude areas perhaps being more likely to suffer from depression due to their physical and mental weaknesses. Therefore, we selected adults aged 60 ≥ years old, according to the definition of older adults in the “Law of the People′s Republic of China on the Protection of the Rights and Interests of the Elderly”, and 594 participants met the selected criteria for age. Of these, 72 older adults aged > 80 years old (advanced age) were excluded due to the small sample size and low representativeness. As such, a total of 522 participants aged 60 to 80 years old remained. Then, 15 participants were excluded due to missing values from 40% or more in regard to the Center for Epidemiological Studies–Depression Scale (CES-D-10). Finally, 507 participants were then included in the analysis. The flow chart of sampling is shown in Figure 1, and the sociodemographic characteristics of the participants are presented in Table 1.

### 2.2. Procedure

This survey aims to understand the physical and mental health of adult Tibetans in China and was conducted by the Institute of Psychology of the Chinese Academy of Sciences and the National Health and Family Planning Commission’s Institute of Science and Technology. The survey combined cluster sampling and convenience sampling; each county was a cluster, and participants included adults from all six county-level administrative regions who were recruited in order to participate in our study via convenience sampling. The Mother and Children Health Hospital of each county was responsible for sending a notice to the township hospitals within its jurisdiction and asking participants to complete the questionnaires at their township hospitals. However, some of the participants in a small number of townships could not be included in our sample. This was due to the fact that their home was far away from the test site and therefore transportation was inconvenient.

All the research assistants received standardized and strict training in order to better supervise the investigation. They introduced the survey purposes and provided prompt instruction to participants during the tests. After a complete description of the survey, the participants were assured that their responses would be kept completely confidential. The trained research assistants administered the questionnaires using the same procedures. All participants completed the questionnaires voluntarily. Written informed consent was obtained from each participant. The survey protocol was approved by the ethics review committee of the Institute of Psychology, Chinese Academy of Sciences (Project identification code: H16014). More detailed information about the participants and procedures was published in a previous study [4].

### 2.3. Measures

Depressive symptoms were assessed using the shortened CES-D-10 [31]. The CES-D-10 scale consists of ten items from the original 20 items Likert scale questionnaire, with response options of 0 (rarely or none of the time) to 3 (most or all of the time). The time frame for assessing depressive symptoms was “during the past week”. Two of the ten items that were positively rated (‘I felt hopeful about the future’ and ‘I was happy’) were reverse scored for the analysis. Total scores ≥10 indicated the presence of significant depression [31]. The Chinese version of the CES-D-10 has been validated and widely used in Chinese adults [32]. In the present study, Cronbach’s alpha value for this measure was 0.82, and the KMO value of confirmatory factor analysis was 0.84.

### 2.4. Statistical Analyses

Descriptive statistical analyses were conducted with SPSS (IBM SPSS, Version 21.0, IBM, Armonk, NY, USA ). Network analysis was conducted using R Core Software, version 4.0.3 (R Team, Vienna, Austria). The packages used included qgraph [33] and bootnet [34].

#### 2.4.1. Network Estimation

Networks are graphical models consisting of nodes and edges. Each node represented a symptom of depression. The edges represent pairwise relationships between these symptoms, after conditioning on all other nodes in the analysis. For the network analyses of depression, we used the qgraph R package [33]. Network models estimating the associations between symptoms were constructed using the Gaussian graphical model (GGM) [34]. GGMs are undirected networks in which the edges represent partial correlation coefficients. To produce a network model with good prediction accuracy, the least absolute shrinkage and selection operator (LASSO) regression method was employed [35]. This technique shrinks all edges, constrains the very small edges to zero, and regularizes the network. Model selection was conducted using the extended Bayesian information criterion (EBIC), and the tuning parameter (λ) was assigned a value of 0.5. Finally, the network was visualized using the Fruchterman–Reingold algorithm [36] in the qgraph R package. The algorithm places nodes with more or stronger connections in the center of the network. In the current study, green and red edges represent positive and negative associations, respectively. Line thickness reflects association strength.

#### 2.4.2. Centrality Estimation

The node centrality metrics include strength, closeness, betweenness, and expected influence (EI). Strength is the sum of the edge weights connected to a node and is considered to be the most stable and reliable measure of node centrality. Recently, EI has been used to assess node centrality instead of strength, especially when negative edges are present. EI reflects the summed weight of positive and negative edges with neighboring nodes in the network [37]. Closeness is the inverse of the sum of the distances of one node from all the others. Betweenness is the number of times that a node lies on the shortest path between two other nodes. A previous study indicated that both closeness and betweenness are not reliably estimated due to being affected by sampling variability and spurious covariance between symptoms [38]. Thus, the symptoms with the highest centrality were examined using EI indices in the current study, and higher values of EI reflect greater node centrality [39]. The indices of node strength, closeness, and betweenness were used for supplementary analyses.

#### 2.4.3. Network Accuracy, Stability, and Significance Testing

To identify the estimated network accuracy and stability, robustness analyses were conducted by using the bootnet R package [34]. First, network accuracy was assessed by determining a nonparametric bootstrap approach. The bootstrapped 95% confidence intervals (CIs) around the edged weights were estimated. Smaller CIs represent greater accuracy in estimation. Second, network stability was examined with the case-dropping subset bootstrap with a correlation stability coefficient (CS-coefficient). The CS-coefficient should not be below 0.25, and a coefficient exceeding 0.50 suggests strong stability and interpretability [34]. Finally, a bootstrapped difference test was conducted in order to examine significant differences between edge weights and node centralities, as well as to show whether they significantly differ from each other in centrality estimates. For this analysis, the interpretation of node centrality differences was based on node strength. This is because estimated node strength has been considered more reliable in previous studies [34,40]. All bootstrap calculations involved 1,000 iterations in the current study.

## 3. Results

### 3.1. Descriptive Analyses

The mean age of all participants was 67.42 (*SD* = 5.61). The mean CES-D-10 total score for all participants was 7.83 (*SD* = 5.13). The proportion of participants who reached the cut-off (≥10) on the CES-D-10 scale was 30.4%. The results of descriptive analyses are shown in Table 1.

### 3.2. Network Structure and Centrality Estimation

**Figure 2:**
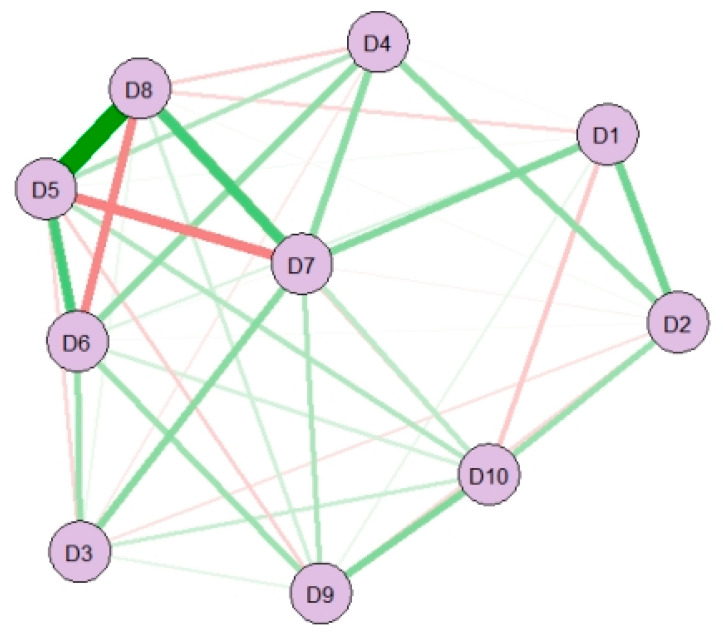
Network structure of depression. Note. D1 = bothered by things; D2 = difficulty keeping mind; D3 = depressed mood; D4 = everything is an effort; D5 = hopelessness about the future; D6 = fear; D7 = poor sleep; D8 = unhappiness; D9 = loneliness; and D10 = could not get going. Nodes represent depression symptoms and edges represent partial correlations between symptoms. Green and red edges represent positive and negative associations, respectively. Line thickness reflects association strength. The stronger and saturated edges represent stronger regularized partial correlations.

A visualization of the network structure of depression can be seen in Figure 2. More edges were estimated to be nonzero in the older adult network (36 of 45 possible edges; density was 0.80). The strongest edge connections emerged between hopelessness about the future and unhappiness (D5:D8), followed by hopelessness about the future and fear (D5:D6), hopelessness about the future and poor sleep (D5:D7), fear and unhappiness (D6:D8), and poor sleep and unhappiness (D7:D8).

**Figure 3:**
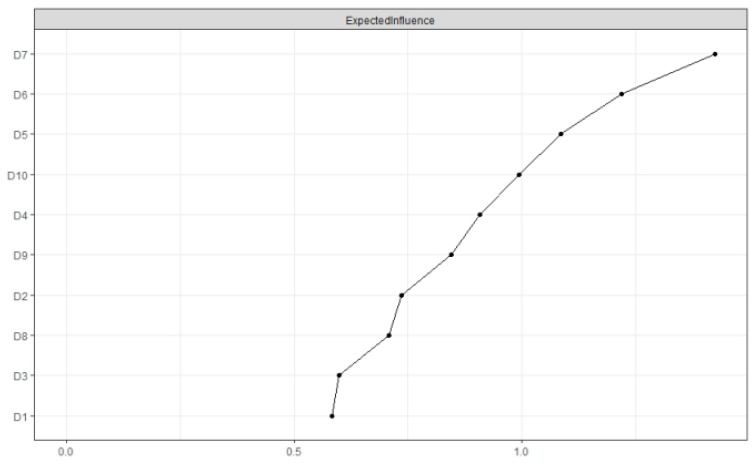
Network-centrality-expected influence for depression. Note. Higher values of expected influence reflect greater node centrality. D1 = bothered by things; D2 = difficulty keeping mind; D3 = depressed mood; D4 = everything is an effort; D5 = hopelessness about the future; D6 = fear; D7 = poor sleep; D8 = unhappiness; D9 = loneliness; and D10 = could not get going.

Network centrality indices for the depression network are depicted in Figure 3. The high central nodes, based on the EI results, were poor sleep (D7), fear (D6), and hopelessness about the future (D5). The standardized estimates of strength, closeness, and betweenness are depicted in Figure S1 in the Supplementary Materials.

### 3.3. Network Accuracy and Stability

The edge-weight bootstrap results revealed that there was considerable overlap between the 95% CIs of the edge weights; however, some of the strongest edges showed non-overlapping CIs in the network, which suggested that network parameters were estimated with good accuracy (Supplementary Figure S2). The CS coefficient for EI was 0.75 in the network (Supplementary Figure S3), reflecting a highly stable network. See Supplementary Figures S4 and S5 for bootstrap significance tests of edge weights and node centrality, respectively.

## 4. Discussion

The current cross-sectional study identified the network structure of depression in older adults living on the Qinghai–Tibet Plateau, the highest plateau in the world. Generally, the results showed that the strongest edge connections emerged between unhappiness and hopelessness about the future, followed by hopelessness about the future and fear, hopelessness about the future and poor sleep, fear and unhappiness, and then poor sleep and unhappiness. Moreover, poor sleep, fear, and hopelessness about the future are the most central symptoms in the network, highlighting their importance in the onset and maintenance of depression in this population. The central role of these symptoms is in contrast to previous findings, which suggest that the most influential symptoms are interest loss, energy loss, and depressed mood in adults [19,25,26], as well as sadness and could not get going in older widowed adults [28]. The discrepancies may result from different participants; previous studies investigated the depression network in youth adults or widowed older people, while the current study identified a network structure of depression in older adults who lived in high-altitude areas.

Specifically, poor sleep emerged as the most central symptom in the network. This finding is in line with previous studies suggests that poor sleep is common in older adults in high-altitude areas [15,41] and is associated with an increased risk of depression [15]. On the one hand, residents living in high-altitude areas have a risk of an apnea–hypopnea index and changed sleep structure [42,43], which may cause poor sleep in older adults; further, a study indicated that poor sleep directly affected individuals’ mood and emotion [44], which may be significant contributors to the development of depression. On the other hand, older people experience some major life events with age, including retirement and bereavement, which may affect social activities and social networks [45,46] and subsequently lead to a lack of sleep [44]. Insufficient or poor sleep reduces life satisfaction [41], and thus increases vulnerability to depression in older adults.

Fear and hopelessness about the future also emerged as symptoms of more importance in the network. A feeling of fear is common in older adults [47,48]. Fear in older people is mostly concerning fear of physical and cognitive decline, dependency, and decreased income. Previous studies have focused on fear of falling, physical illness (e.g., cancer), or crime in old age [49,50]. Further, it has been indicated that fear may restrict outdoor activities and social contact, and thus may increase the risk of depression [50]. Moreover, later life can be more unpredictable than other stages in life, which may cause more feelings of fear and hopelessness about the future. Older people fear more about an unknown future, including fear of dying or being left alone after their partner has died [47]. Facing unknown and pessimistic views about the future leads to a further sense of hopelessness about the future, which could increase the likelihood of experiencing negative emotions and further contribute to the development of depression [51]. Additionally, the participants in this study were from high-altitude areas, which are low economic areas in China. Furthermore, the older adults had a lower monthly income. Financial difficulties for older adults will increase the burden of treatment, cause more fear regarding physical conditions [52], and trigger a sense of hopelessness about the future, which may accelerate the formation of depression in older adults.

Finally, the strongest connection emerged between unhappy and hopelessness about the future, in the network. This result suggests the possibility that feeling unhappiness may foster hopelessness about the future, and hopelessness about the future may foster feelings of unhappiness. A previous study indicated that different perceptions of the future may be explained by people’s past and present experiences [47]. The harsh natural environment (e.g., cold climate, strong winds, etc.) and the poor economic condition of high-altitude areas may bring uncomfortable feelings to the body and mind of older adults in the past and present, which may, therefore, trigger unhappiness. This negative physical and mental experience further leads to a sense of hopelessness about the future. In addition, we found a stronger connection between hopelessness about the future and fear, hopelessness about the future and poor sleep, fear and unhappiness, and poor sleep and unhappiness, which may reflect mutually reinforcing feedback loops [53]. Neuropsychological studies have found that high-altitude exposure leads to impairment of human executive function [54]. Executives function as conductors that have important roles in controlling, organizing, and directing cognitive activity, emotional responses, and behavior [55]. Executive impairment increases the risk of depressive symptoms [56], such as fear, unhappiness, and hopelessness about the future. Moreover, older adults have less awareness and focus less attention to mental health. Further, the limited mental health service in high-altitude areas causes bidirectional relations between these symptoms, and is likely to increase vulnerability to the development and maintenance of the depression network.

The present study had several limitations of note. First, the node centrality and self-reinforcing edges were calculated with cross-sectional data, which does not allow exploration of dynamic processes within symptom networks. Future evidence could include longitudinal data in order to evaluate the changes in the network structure of depression across long time periods. Second, depressive symptoms were self-reported rather than obtained through a clinical assessment. Future studies should assess the network structures of depression based on clinical observations in order to replicate and validate our findings. Third, the current study only included older adults living in high-altitude areas. Future studies should consider adding a matched group from low-altitude areas to further identify the impact of high altitude on depression. Last, considering the length of the manuscript, the current study does not discuss many more possible mediators and moderating factors, which should be considered in future studies.

Despite these limitations, the present study has important practical implications for both researchers and clinicians working in this area. Specifically, we identified that symptoms with high centrality were poor sleep, fear, and hopelessness about the future in older adults. This suggests that these symptoms may be the most important targets for intervention, as it is possible that direct interventions addressing symptoms with high centrality are more beneficial in order to assist with recovery from depression. For instance, improving sleep quality, reducing fear, and enhancing the sense of hope for the future in older people by providing material and spiritual support may be beneficial to the recovery from depression in these populations. In addition, the strongest connection between unhappiness and hopelessness about the future indicated that these symptoms may also be vital targets for treatment as modifying edge (symptom–symptom) correlations can change the network structure itself and thus achieve the effect of network intervention [53]. The network identified in this study suggests that building a sense of hope and safety for the future could reduce the widespread influence of negative moods in depressive symptoms. It is worth noting that interveners should consider the network structure features in order to capture information about the characteristics of depression before implementing an intervention; further, they should consider selecting an appropriate psychological intervention technique.

## 5. Conclusions

The present study applied network analysis in order to identify the symptom structure of depression among older adults on the Qinghai–Tibet Plateau. The findings highlight the importance of poor sleep, fear, and hopelessness about the future in older adults’ depression. The strongest edge connections emerged between unhappy and hopelessness about the future, followed by hopelessness about the future and fear, hopelessness about the future and poor sleep, fear and unhappiness, and poor sleep and unhappiness in the networks. These findings provide novel insights into depression in older adults living in high-altitude areas.

## Figures and Tables

**Table 1:** Sociodemographic characteristics of the participants (*n* = 507).

Sociodemographic Characteristic	Category	*n*	%
^a^ Sex	Male	199	39.3
	Female	300	59.2
^a^ Education level	Never went to school	368	72.6
	Primary school	78	15.4
	Junior high school	37	7.3
	High school or above	18	3.6
^a^ Marital status	First marriage	441	87.0
	^b^ Other	57	11.2
^a^ Marriage satisfaction	Dissatisfied	8	1.6
	Neutral	23	4.5
	Satisfied	438	86.4
^a^ Monthly family income (RMB)	≤2000	235	46.4
	>2000	254	50.0
^a^ Number of people living together	1–3	249	49.1
	4–6	226	44.6
	7–12	31	6.1
CES-D-10 total score	≥10	154	30.4
	<10	353	69.6
		*M*	*SD*
Age	-	67.42	5.61
CES-D-10 total score	-	7.83	5.13

Note. ^a^: There are missing values in sociodemographic characteristics; thus, the sum of the effective percentage is not equal to 100% in these cases. ^b^: Other, including single/divorced/separated/widowed.

## Data Availability

The datasets generated and analyzed during the current study are not publicly available as to ensure the privacy of participants, but are available from the corresponding authors upon reasonable request.

## Supplementary Materials

The following supporting information can be downloaded at: https://www.mdpi.com/article/10.3390/ijerph192113810/s1, Figure S1. Strength, closeness, and betweenness estimates for the depression network. Figure S2. Bootstrapped 95% confidence intervals of the estimated edge weights in the depression network. Figure S3. Case-dropping subset bootstrap for the depression network. Figure S4. Bootstrapped difference tests of edge weights of the depression network. Figure S5. Bootstrapped difference tests of node strength of the depression network.
