# Supplementary material for: Symptom Structure of Depression in Older Adults on the Qinghai–Tibet Plateau: A Network Analysis
Source: Int J Environ Res Public Health. 2022 Oct 24;19(21):13810. doi: 10.3390/ijerph192113810 (PMC9659106; doi:10.3390/ijerph192113810)

Symptom Structure of Depression in Older Adults on the Qinghai–Tibet Plateau: A Network Analysis

Supplemental materials

Included in this file are:

- *Figure S1.* Strength, closeness, and betweenness estimates for the depression network.
- *Figure S2.* Bootstrapped 95% confidence intervals of the estimated edge weights in the depression network.
- *Figure S3.* Case-dropping subset bootstrap for the depression network.
- *Figure S4.* Bootstrapped difference tests of edge weights of the depression network.
- *Figure S5.* Bootstrapped difference tests of node strength of the depression network.

Figure S1. Strength, closeness, and betweenness estimates for the depression network.

Note. Higher values of expected influence reflect greater node centrality.

D1 = bothered by things; D2 = difficulty keeping mind; D3 = depressed mood; D4 = everything is an effort; D5 = hopelessness about future; D6 = fear; D7 = poor sleep; D8 = unhappiness; D9 = loneliness; D10 = could not get going.

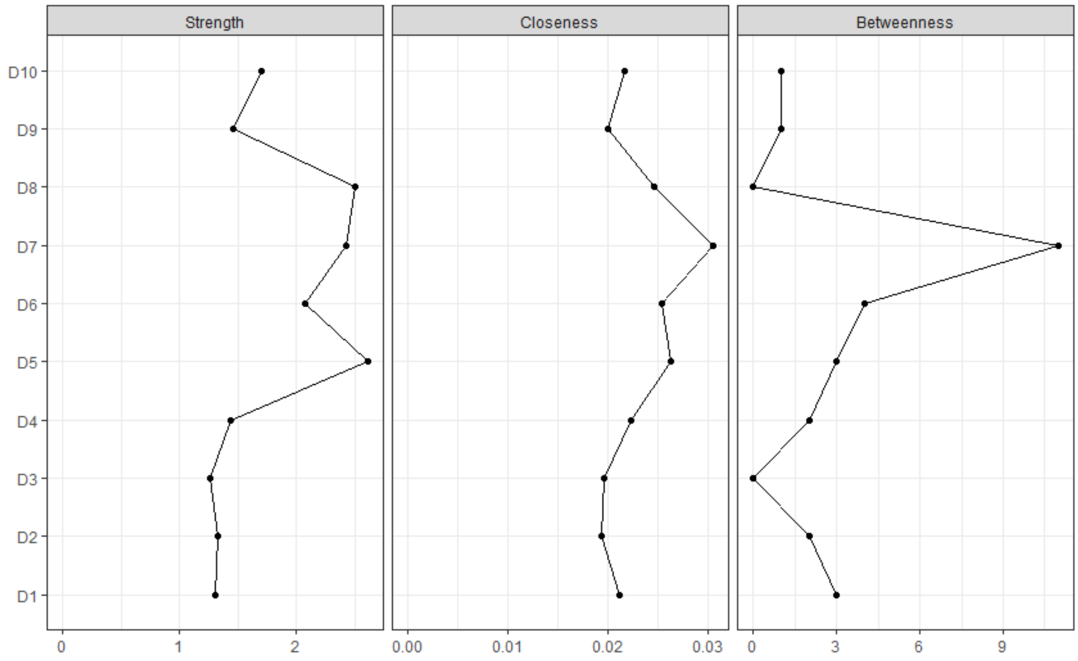

Figure S2. Bootstrapped 95% confidence intervals (CIs) of the estimated edge weights in the depression network.

Note. The red line indicates the sample edge weight values, the black line represents bootstrap means, and the gray area indicates the 95% CIs. Each horizontal line represents one edge of the network, ordered from the edge.

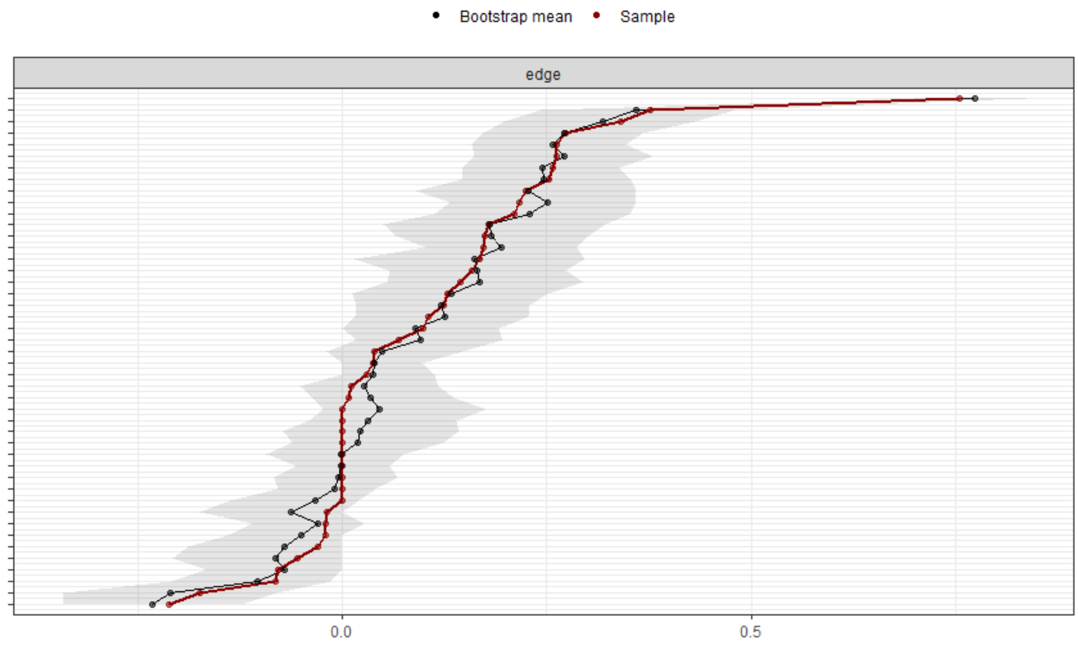

Figure S3. Case-dropping subset bootstrap for the depression network.

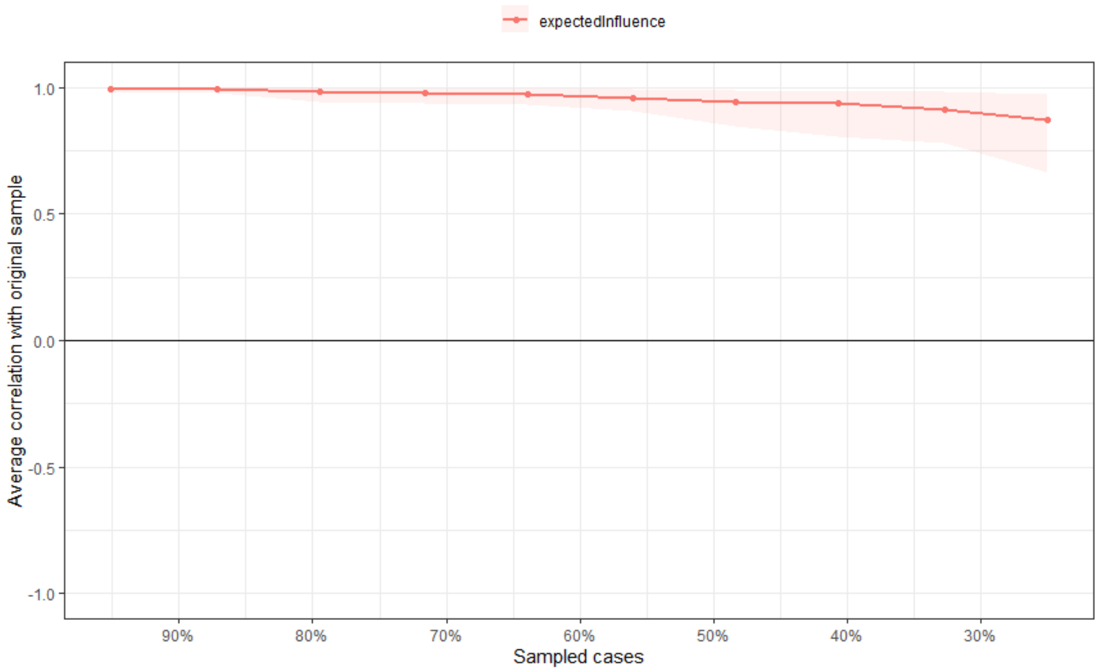

Figure S4. Bootstrapped difference tests of edge weights of the depression network.

Note. Black boxes indicate a significant difference between two edges (α = .05). Grey boxes indicate no significant difference.

V1 = bothered by things; V2 = difficulty keeping mind; V3 = depressed mood; V4 = everything is an effort; V5 = hopelessness about future; V6 = fear; V7 = poor sleep; V8 = unhappiness; V9 = loneliness; V10 = could not get going.

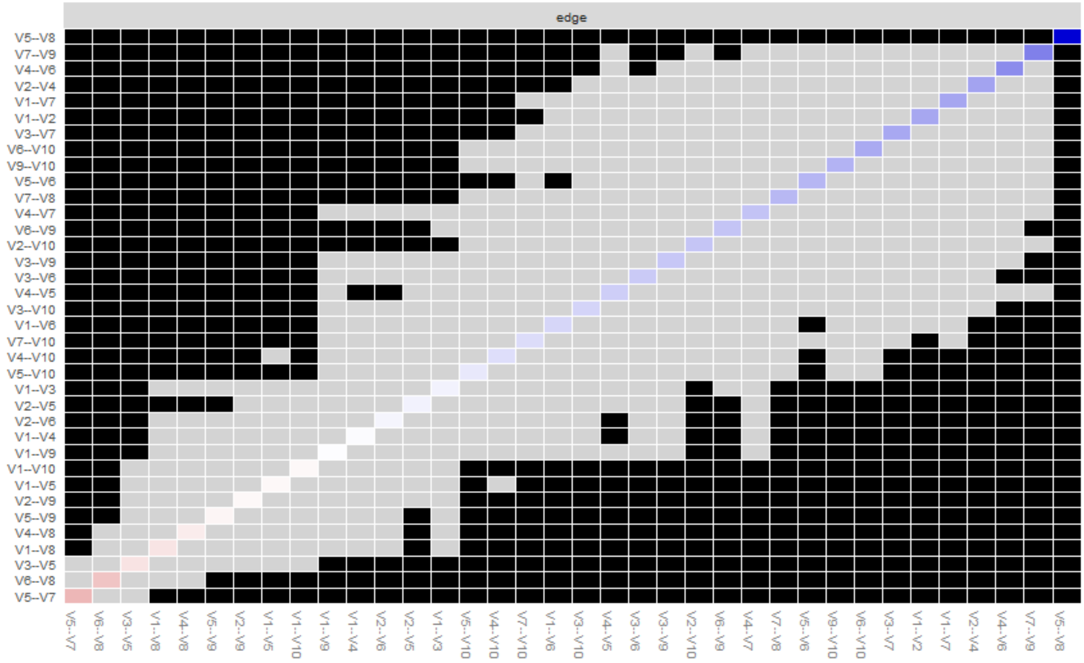

Figure S5. Bootstrapped difference tests of node strength of the depression network.

Note. Black boxes indicate a significant difference between two nodes (α = .05). Grey boxes indicate no significant difference.

V1 = bothered by things; V2 = difficulty keeping mind; V3 = depressed mood; V4 = everything is an effort; V5 = hopelessness about future; V6 = fear; V7 = poor sleep; V8 = unhappiness; V9 = loneliness; V10 = could not get going.

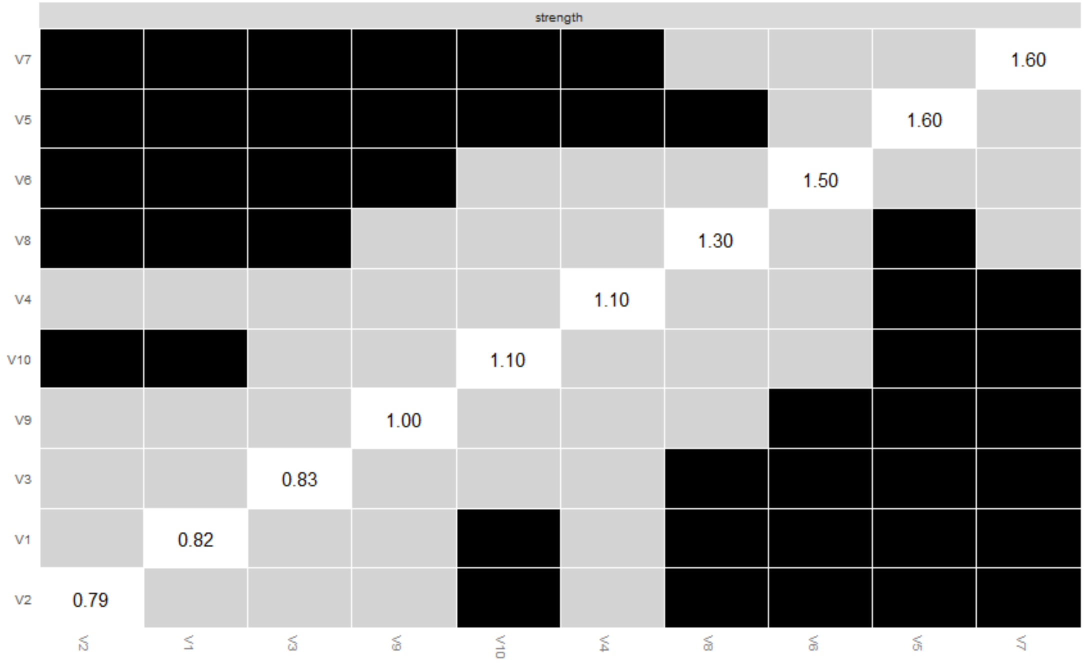

Supplement: Supplementary file 1 [file ijerph-19-13810-s001.zip › ijerph-1971456-supplementary.pdf]
